# Effect of different revascularization times on intermediate-risk non-ST-elevation acute coronary syndrome

**DOI:** 10.1038/s41598-022-20185-9

**Published:** 2022-09-20

**Authors:** Xiangyong Kong, Jun Yin, Hongwu Chen, Jiawei Wu, Xiaofan Yu, Ningtian Zhou, Likun Ma

**Affiliations:** 1 Division of Life Sciences and Medicine, Department of Cardiology, The First Affiliated Hospital of USTC, University of Science and Technology of China, Hefei, 23001 Anhui China; 2 Department of Cardiology, The First Affiliated Hospital of Nanjing Medical University, Nanjing, Jiangsu China

**Keywords:** Cardiac device therapy, Interventional cardiology

## Abstract

Non-ST-elevation acute coronary syndrome (NSTE-ACS) is a specific type of acute coronary syndrome. We applied the Thrombolysis in Myocardial Infarction (TIMI) score for risk stratification of patient prognosis. There was uncertainty about the routine revascularization time in patients with intermediate-risk NSTE-ACS. A total of 2835 patients with intermediate-risk NSTE-ACS (TIMI score 3–4) included in the China Acute Myocardial Infarction Registry from November 2014 to January 2017 were analyzed according to the time window from symptom onset to revascularization: within 24 h, Group I (814/28.7%); within 24 to 48 h, Group II (526/18.6%); within 48 to 72 h, Group III (403/14.2%); and after 72 h, Group IV (1092/38.5%). Risk factors, management and in-hospital outcomes were analyzed in the four groups. The results of the chi-square test showed that there was a significant difference in the incidence of in-hospital major adverse cardiovascular events (MACEs) when revascularization was completed within 48 h than when it was completed after 48 h (P < 0.05). The results of revascularization within 48 h were similar, and the incidence of in-hospital MACEs was lower than when revascularization was completed after 48 h. The incidence of in-hospital MACEs among patients who underwent revascularization within 48 h is lower than that of patients who underwent revascularization after 48 h.

## Introduction

Non-ST-elevation acute coronary syndrome (NSTE-ACS) is a specific type of acute coronary syndrome that often presents with ischemic chest pain but does not show typical ST-segment elevation on electrocardiogram. The pain that occurs in acute coronary syndrome is often caused by thrombus obstruction, and the thrombus that appears when myocardial infarction occurs is currently considered to be a white thrombus, with platelets as the main component, which does not allow thrombolysis to solve the problem. Percutaneous coronary intervention (PCI) treatment is still the main treatment option. Currently, the incidence of non-ST-elevation acute coronary syndrome is almost twice that of ST-elevation acute coronary syndrome^1^. Therefore, we need to pay more attention to what is relevant in the context of follow-up treatment. Patients with NSTE-ACS are at risk for adverse cardiac events because of refractory angina and hemodynamic or electrical instability^2,3^. A routine early invasive strategy of early angiography followed by revascularization was compared with a conservative strategy of angiography and subsequent revascularization, which was performed only in patients in whom drug therapy failed or those with substantial residual ischemia. An early invasive strategy of coronary angiography was favored, especially in high-risk subgroups, and this strategy has been shown to be beneficial in many studies^3,4^. As a result, the latest guidelines from the American College of Cardiology-American Heart Association and the European Society of Cardiology recommended that an early invasive approach should be performed within 24 h in high-risk NSTE-ACS patients and within 72 h in intermediate-risk NSTE-ACS patients^5,6^. However, the optimal timing of this intervention for intermediate-risk patients has not been well defined. Current studies have shown no significant difference between early invasive treatment and immediate invasive or delayed invasive approaches, but these studies often start at the time of admission, which may lead to significant heterogeneity in the trial cohort in terms of timing intervals, so we planned the starting point of this study to be at the onset of symptoms to reduce the impact of such issues^7–9^. Additionally, based on the data in the CCC (Improving Care for Cardiovascular Disease in China), we decided to analyze the prognosis according to the timing of PCI in patients with intermediate-risk NSTE-ACS in China. The primary purpose of our study was to first select intermediate-risk NSTE-ACS patients. Thereafter, we grouped patients according to the time interval from the onset of cardiac symptoms to PCI. Then, we reviewed their in-hospital clinical outcomes.

## Materials and methods

### Study design

The CCC-ACS project is a nationwide registry and quality improvement study with a constantly updated database placing emphasis on ACS care. This study was jointly initiated by the American Heart Association and the Chinese Society of Cardiology in 2014. The study design and methodological specifics of the project have been published^10^. Clinical Trial Registration URL: http://www.clinicaltrials.gov. Unique identifier: NCT02306616. All methods were performed in accordance with the relevant guidelines and regulations.

### Study population

We obtained information on the study population from the Chinese Acute Myocardial Infarction Registry, and after considering the completeness of the information, we selected all patients with NSTE-ACS from November 2014 to January 2017. Patients with a score of 3 to 4 were screened according to the TIMI (Thrombolysis in Myocardial Infarction) score^11^. We pooled and retrospectively analyzed all eligible patients undergoing PCI and finally divided them into 4 groups according to the time interval from the onset of cardiac symptoms to PCI. In total, 814 patients (28.7%) in whom revascularization was completed within 24 h were allocated to Group I, 526 patients (18.6%) in whom revascularization was completed within 24 to 48 h were allocated to Group II, 403 patients (14.2%) in whom revascularization was completed within 48 to 72 h were allocated to Group III, and 1092 patients (38.5%) in whom revascularization was completed after 72 h were allocated to Group IV. Institutional review board approval was granted for this research by the ethics committee of Beijing Anzhen Hospital, Capital Medical University. Informed consent for participation was obtained from all patients.

### Medical treatment and collection of clinical data

The patients received 150 to 300 mg of aspirin at the time of admission, followed by at least 75 to 100 mg of aspirin daily for an indefinite period; 300 to 600 mg of clopidogrel or 180 mg of ticagrelor was also immediately given, with 75 mg of clopidogrel or 180 mg (90 mg bid) of ticagrelor taken daily thereafter. Angiotensin-converting enzyme inhibitors, angiotensin-receptor blockers, glycoprotein IIb/IIIa inhibitors, statins, unfractionated heparin, low-molecular-weight heparin and beta-blockers were administered according to the attending doctor’s decision. The clinical information included patient characteristics, medical history, laboratory results, angiography findings, and outcomes during hospitalization.

### Statistical analysis

Categorical variables are described as frequencies and percentages. Continuous variables are presented as the mean ± SD or medians (interquartile range) according to different distributions. The chi-square test was used to assess the associations between risk factors and different reperfusion time groups. Multiple logistic regression analyses and the chi-square test were used to adjust for the following factors affecting in-hospital MACEs: age; baseline comorbidities, such as diabetes mellitus, hypertension, prior myocardial infarction (MI), chronic obstructive pulmonary disease (COPD), congestive heart failure (CHF), and peripheral arterial disease (PAD); a history of PCI/coronary artery bypass grafting (CABG); and the use of aspirin, beta-blockers, angiotensin-receptor blockers, or lipid-lowering agents. All statistical tests were 2-tailed, and a P value < 0.05 was considered statistically significant. All statistical analyses were conducted with SPSS 23.0 (IBM).

### Ethics approval and consent to participate

The study was approved by the Committee of The First Affiliated Hospital of University of Science and Technology of China.

## Results

### Study population

Table 1 lists baseline characteristics that were similar, excluding age, sex, prior CHF and hypertension, among groups. Significant differences in peak troponin I, peak CK-MB and NT-ProBNP were observed (P < 0.05). Troponin I and CK-MB peaked at different times, reflecting different time periods of myocardial injury. In terms of medications, significant differences in clopidogrel, GP IIb/IIIa inhibitor and anticoagulant use were observed (P < 0.05). There were no serious complications except peripheral subcutaneous hematoma during the operation; therefore, procedural complications were not included in the baseline characteristics.

**Table 1:** Baseline characteristics of patients.

Number (%)/Mean (STD)	Overall (n = 2835)	Group I (< 24 h) n = 814 (28.7%)	Group II (24–48 h) n = 526 (18.6%)	Group III (48–72 h) n = 403(14.2%)	Group IV (> 72 h) n = 1092(38.5%)	P-value
**Demographic characteristics**						
Age (years)	62.91 ± 11.05	61.66 ± 11.23	63.25 ± 11.11	62.5 ± 11.03	63.82 ± 10.82	< 0.001
Male (%)	2107(74.3)	634(77.9)	390(74.1)	287(71.2)	796(72.9)	0.035
**Medical history**						
Current smoker	1122(39.6)	348(42.8)	209(39.7)	156(38.7)	409(37.5)	0.132
Prior MI	245(8.6)	74(9.1)	45(8.6)	36(8.9)	90(8.2)	0.924
Prior PCI	334(11.8)	93(11.4)	74(14.1)	54(13.4)	113(10.3)	0.116
Prior CABG	10(0.4)	0(0)	1(0.2)	3(0.7)	6(0.5)	0.101
Prior CHF	36(1.3)	6(0.7)	2(0.4)	7(1.7)	21(1.9)	0.022
COPD	53(1.9)	10(1.2)	7(1.3)	12(3)	24(2.2)	0.112
PAD	39(1.4)	16(2)	7(1.3)	5(1.2)	11(1)	0.356
Hypertension	1677(59.2)	455(55.9)	304(57.8)	238(59.1)	680(62.3)	0.039
dyslipidemia	332(11.7)	87(10.7)	58(11)	60(14.9)	127(11.6)	0.171
Diabetes	651(23)	167(20.5)	127(24.1)	91(22.6)	266(24.4)	0.221
Renal insufficiency	45(1.6)	9(1.1)	7(1.3)	6(1.5)	23(2.1)	0.342
Cerebrovascular disease	269(9.5)	61(7.5)	48(9.1)	40(9.9)	120(11)	0.078
Family history (CHD)	77(2.7)	28(3.4)	16(3)	8(2)	25(2.3)	0.335
**Laboratory results (mean ± STD)**						
Peak Troponin I (ug/L)	3.74 ± 9.21	4.5 ± 10.49	2.8 ± 7.12	2.17 ± 5.79	4.17 ± 9.93	0.001
Peak CKMB (U/L)	31.61 ± 71.29	43.59 ± 107.31	23.84 ± 51.75	25.97 ± 43.31	28.01 ± 46.55	< 0.001
Scr (mg/dl)	82.93 ± 52.94	79.83 ± 49.88	81.24 ± 43.22	82.72 ± 53.37	86.1 ± 58.77	0.071
NT pro-BNP	1226.12 ± 2706.84	1006.23 ± 2473.21	780.06 ± 1541.19	1156.19 ± 2178.84	1670.09 ± 3415.03	< 0.001
TC (mg/dl)	4.39 ± 1.22	4.43 ± 1.21	4.34 ± 1.34	4.33 ± 1.15	4.41 ± 1.19	0.398
HDL-C (mg/dl)	1.06 ± 0.33	1.07 ± 0.37	1.04 ± 0.3	1.07 ± 0.36	1.07 ± 0.31	0.356
LDL-C (mg/dl)	2.69 ± 0.97	2.74 ± 1.02	2.63 ± 0.96	2.68 ± 0.95	2.7 ± 0.95	0.321
TG (mg/dl)	1.86 ± 1.57	1.87 ± 1.5	1.94 ± 1.98	1.79 ± 1.36	1.83 ± 1.47	0.45
**Medications**						
Aspirin	2697(95.1)	783(96.2)	509(96.8)	381(94.5)	1024(93.8)	0.068
Clopidogrel	2445(86.2)	670(82.3)	458(87.1)	350(86.8)	967(88.6)	0.001
Beta-blockers	1679(59.2)	461(56.6)	314(59.7)	245(60.8)	659(60.3)	0.425
ACE-inhibitors/ARB	1477(52.1)	433(53.2)	282(53.6)	189(46.9)	573(52.5)	0.151
Statins	2706(95.4)	771(94.7)	508(96.6)	386(95.8)	1041(95.3)	0.72
GP IIb/IIIa inhibitor	682(24.1)	259(31.8)	124(23.6)	87(21.6)	212(19.4)	< 0.001
Anticoagulant	2022(71.3)	613(75.3)	366(69.6)	269(66.7)	774(70.9)	0.01

*MI* myocardial infarction, *PCI* percutaneous coronary intervention, *CABG* coronary artery bypass graft surgery, *CHF* congestive heart failure, *PAD* peripheral artery disease, *CHD* coronary heart disease, *ACEI* angiotension converting enzyme inhibitor, *ARB* angiotension receptor blocker.

### Target vessels of PCI

Table 2 shows the extent of coronary disease in target vessels, and there were no significant differences, excluding coronary disease of 1 or 2 vessels.

**Table 2:** Coronary angiographic characteristics.

	Overall	Group I (< 24 h)	Group II (24–48 h)	Group III (48–72 h)	Group IV (> 72 h)	P-value
Total	2835	814	526	403	1092	
**Extent of coronary disease (n, %)**						
1-vessel disease	2182(80.5)	655(86)	399(78.4)	312(79.6)	816(78)	0.028
2-vessel disease	348(12.8)	71(9.3)	76(14.9)	49(12.5)	152(14.5)	0.002
3-vessel disease	179(6.6)	36(4.7)	34(6.7)	31(7.9)	78(7.5)	0.057
**Target vessel (n, %)**						
LM	112(4)	25(3.1)	24(4.6)	9(2.2)	54(4.9)	0.043
LAD	1605(56.6)	424(52.1)	304(57.8)	226(56.1)	651(59.6)	0.011
LCX	824(29.1)	220(27)	156(29.7)	123(30.5)	325(29.8)	0.493
RCA	986(34.8)	261(32.1)	193(36.7)	154(38.2)	378(34.6)	0.133

*LM* left main coronary artery, *LAD* left anterior descending, *LCX* left circumflex artery, *RCA* right coronary artery.

### In-hospital outcomes

**Figure 1:**
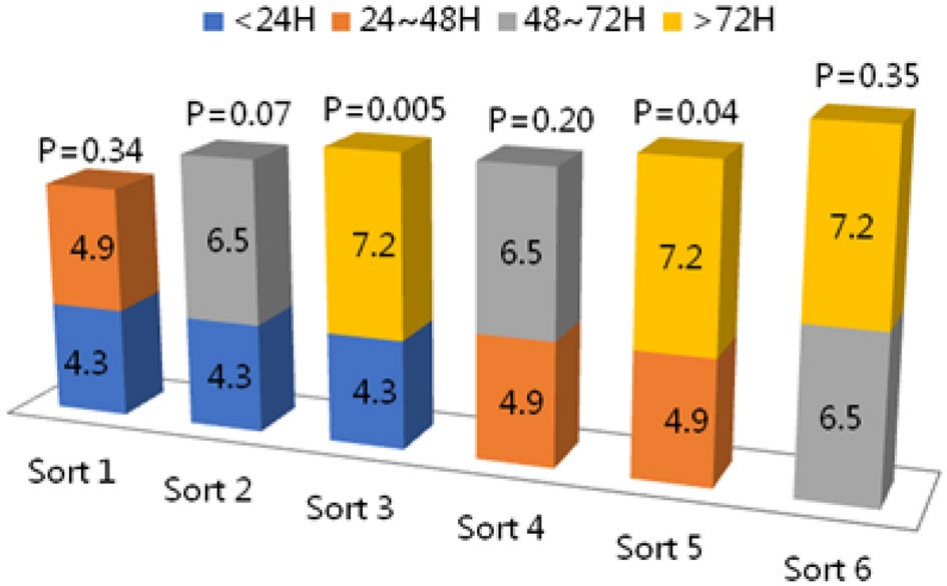
Comparison of MACE event rates among groups. Different interventional times affect MACE event rates. Sort 2, Sort 3 and Sort 5 showed significant differences (P = 0.07, 0.005, 0.04). Others showed nonsignificant difference (P > 0.05).

In-hospital complications and mortality are summarized in Table 3. Major adverse cardiovascular events (MACEs), including cardiac death, heart failure, cardiogenic shock, ischemic stroke, and bleeding, were noted. The MACEs were compared among groups. The results showed no significant differences in the individual adverse events among groups (P > 0.05), but multivariate logistic regression analysis confirmed that the total incidence of MACEs was higher in Group IV than in Groups I, II, and III (Table 4). The results of the chi-square test showed that there was a significant difference in the incidence of MACEs within 72 h and after 72 h (P < 0.05). There was also a statistically significant difference in the incidence of MACEs within 48 h and after 48 h (P < 0.05, Table 5), but there was no significant difference between Group III and Group IV or between Group I and Group II (P > 0.05, Fig. 1).

**Table3:** In-Hospital outcome.

Major adverse events	Overall	Group I (< 24 h) (%)	Group II (24–48 h) (%)	Group III (48–72 h) (%)	Group IV (> 72 h) (%)	P-value
Total	2835	814	526	403	1092	
Death	5(0.2)	2(0.2)	1(0.2)	1(0.2)	1(0.1)	0.851
Cardiac death	5(100)	2(100)	1(100)	1(100)	1(100)	0.881
Congestive heart failure	128(4.5)	27(3.3)	19(3.6)	20(5)	62(5.7)	0.062
Cardiogenic shock (killip IV)	24(0.8)	9(1.1)	7(1.3)	4(1)	4(0.4)	0.156
Stroke	10(0.4)	3(0.4)	0(0)	0(0)	7(0.6)	0.118
Hemorrhagic stroke	8(80)	3(100)	0(0)	0(0)	5(71.4)	0.187
Major bleeding	43(1.5)	11(1.4)	7(1.3)	4(1)	18(1.6)	0.913

**Table 4:** Multivariate logistic regression analysis of in-hospital MACEs.

Group	MACE (%)	Crude OR (95% CI)	Adjusted OR^a^ (95% CI)	Adjusted OR^b^ (95% CI)	Adjusted OR^c^ (95% CI)
PCI < 24 h	35(4.3)	1	1	1	1
PCI 24–48 h	26(4.9)	1.157(0.688–1.946)	1.108(0.658–1.867)	1.149(0.678–1.947)	1.286(0.751–2.202)
PCI 48–72 h	26(6.5)	1.535(0.911–2.587)	1.506(0.892–2.544)	1.457(0.853–2.489)	1.719(0.99–2.987)
> 72 h	79(7.2)	1.736(1.153–2.612)	1.646(1.092–2.482)	1.546(1.016–2.352)	1.918(1.246–2.953)

Adjusted for variables that Stepwise Multiple Logistic regression p-remove > 0.2 and p-enter < 0.1.

^a^sex, age.

^b^Sex, age, smoking, diabetes mellitus, hypertension, dyslipidemia, renal insufficiency, prior MI, PCI, CABG, COPD, CHF, PAD.

^c^Sex, age, smoking, diabetes mellitus, hypertension, dyslipidemia, renal insufficiency, prior MI, PCI, CABG, COPD, CHF, PAD, ASA, clopidogrel, beta-blocker, statin, angiotensin converting enzyme inhibitor, GP IIb/IIIa antagonist, anticoagulant drugs.

**Table 5:** Incidence of in-hospital MACEs between PCI < 48 h and ≥ 48 h.

Group	N	Non- MACE (%)	MACE (%)	*X*^2^	P-value
PCI < 48 h	1340	1279 (95.4)	61 (4.6)		
≥ 48 h	1495	1390 (93.0)	105 (7.0)		
Total	2835	2669 (94.1)	166 (5.9)	7.386	0.007

We also investigated the status and syndrome of patients in the process of admission and angiography, and the number of patients receiving different therapies in the 4 groups is illustrated in Table S1.

## Discussion

Many studies have shown that a selective invasive strategy after conservative treatment obtains better results in low-risk NSTE-ACS patients. Additionally, an early invasive strategy has been shown to be beneficial compared to a conservative strategy, especially in high-risk subgroups of patients with elevated cardiac troponin levels^5,12^. Therefore, the latest guidelines from the American College of Cardiology-American Heart Association and the European Society of Cardiology recommended an early (< 24 h) invasive strategy for high-risk patients with NSTE-ACS, a < 72-h invasive strategy for intermediate-risk patients with NSTE-ACS, and a selective invasive strategy for low-risk patients with NSTE-ACS^13^.

The optimal time of invasive treatment for high-risk NSTE-ACS patients was well defined, and for low-risk patients, the decision regarding invasive treatment depended on the situation^13^. However, patients with intermediate-risk NSTE-ACS have a wide time window for interventional treatment, which can be initiated within 72 h^2^. It is not clear how to evaluate the optimal intervention time, as there is no unified standard for the timely adjustment of the treatment strategy when the patient's condition changes during hospitalization^14,15^.

Therefore, on the basis of previous studies^16–18^, according to TIMI risk score, we mainly selected the most representative intermediate-risk NSTE-ACS patients for our study. The prognostic value of both the GRACE risk score and the TIMI risk index has been demonstrated in patients with NSTEMI. In the Chinese population, the TIMI score was shown to be better than the GRACE score in predicting MACEs in NSTEMI patients^19^. Compared to the GRACE risk score, the TIMI risk index was easier to evaluate and could be scored with fewer parameters, so we chose the latter for risk assessment. Then, these patients were divided into four groups according to different PCI time intervals. We analyzed the effect of different interventional times on in-hospital adverse events. Table 1 shows that the baseline characteristics of the patients were similar in the different groups. There was no significant difference among groups in medical history/comorbidity that might affect perioperative prognosis. Thus, the influence of confounding factors on adverse outcomes during hospitalization was avoided, ensuring that the results were rigorous. In intermediate-risk NSTE-ACS patients, our results showed that there was no significant difference in the incidence of individual adverse events among the groups with different interventional times (P > 0.05, Table 3). However, there was a certain correlation between different interventional times and clinical adverse events. Through multivariate logistic regression analysis, our study showed that earlier (< 72 h) interventional times were better than those initiated beyond 72 h, as the risk of in-hospital MACEs was minimized. These results were similar to those of a previous study^13^. Our results suggested that patients in whom revascularization was completed within 48 h had a significantly lower incidence of in-hospital MACE events than patients in whom revascularization was completed after 48 h (Table 5). Additionally, compared to patients who underwent revascularization after more than 72 h, patients in whom revascularization was completed at 48–72 h had no significant difference in the incidence of in-hospital MACEs, but there was a significantly lower incidence of in-hospital MACEs when revascularization was completed within 48 h (Fig. 1). This result suggests that the time of interventional therapy has no significant effect on the incidence of in-hospital MACEs when the onset of cardiac symptoms in intermediate-risk NSTE-ACS patients exceeds 48 h.

### Limitations of the study

A limitation of our study was that it was based on only registry data, not on randomized controlled subjects. Only in-hospital outcomes were evaluated, and the number of patients was small and may be insufficient to discover the true risks of premature coronary heart disease from an individual and societal perspective. Further clinical analysis on a larger population and prospective randomized controlled studies are necessary to ascertain whether using early invasive procedures in patients with intermediate-risk NSTE-ACS leads to better outcomes than late procedures.

## Conclusions

For intermediate-risk NSTE-ACS patients, the incidence of in-hospital MACEs presented an insignificant difference according to the timing of PCI based on the data in China. However, revascularization after 48 h is associated with poor in-hospital outcomes and a significantly different incidence of adverse reactions compared to revascularization within and beyond 48 h in such intermediate-risk NSTE-ACS patients. Further studies are required to assess the optimal revascularization time in this population.

## Supplementary Information

Supplementary Information.

## Data Availability

The datasets used and/or analyzed during the present study are available from the corresponding author on reasonable request.
