# Supplementary material for: Effect of different revascularization times on intermediate-risk non-ST-elevation acute coronary syndrome
Source: Sci Rep. 2022 Sep 20;12:15714. doi: 10.1038/s41598-022-20185-9 (PMC9489762; doi:10.1038/s41598-022-20185-9)
Supplement: Supplementary file 1 — Supplementary Information. [file 41598_2022_20185_MOESM1_ESM.docx]

**Supplementary data**

Table S1 described the syndrome in process of admission and in operation, as well as the ratio of therapeutic choices in 4 groups. In admission, the patients with chest pain status, low blood pressure was significantly different (P＜0.05) in all groups, and ventricular arrhythmia was no significantly different（P＞0.05）. Meanwhile, in angiography, the syndromes including ventricular arrhythmia, coronary spasm, angina pectoris were significantly different (P＜0.05). Indeed, because of the different status of patients in admission, the different therapeutic choices executed in different groups. PCI and CABG were significantly different in all groups(P＜0.05), but conservative treatment was no significantly different（P＞0.05）.

**Table S1. Syndrome & therapy in patients**

| **Group（n）** | **Admission Syndrome（%）** | | | **Angiography Syndrome（%）** | | | | **Therapy（%）** | | |
| --- | --- | --- | --- | --- | --- | --- | --- | --- | --- | --- |
|  | **chest pain status** | **ventricular arrhythmia** | **low blood pressure** | **No reflow** | **ventricular arrhythmia** | **coronary spasm** | **angina pectoris** | **PCI** | **CABG** | **Conservative treatment** |
| **I（814）** | 652（80.1） | 196（24.1） | 98（12.0） | 12（1.5） | 35（4.3） | 67（8.2） | 20（2.5） | 655（80.5） | 20（2.5） | 139（17.0） |
| **II（526）** | 421（80.0） | 101（19.2） | 67（12.7） | 23（4.4） | 32（6.1） | 65（12.4） | 21（4.0） | 408（77.6） | 12（2.3） | 106（20.1） |
| **III（403）** | 333（82.6） | 96（23.8） | 40（9.9） | 31（7.7） | 40（9.9） | 44（10.9） | 31（7.7） | 290（72.0） | 32（7.9） | 81（20.1） |
| **IV（1092）** | 571（52.3） | 232（21.2） | 88（8.1） | 50（4.6） | 109（10.0） | 201（18.4） | 198（18.1） | 789（72.3） | 101（9.2） | 202（18.5） |
| **P** | ＜0.001 | 0.134 | 0.007 | ＜0.001 | ＜0.001 | ＜0.001 | ＜0.001 | ＜0.001 | ＜0.001 | 0.442 |

**PCI: Percutaneous Transluminal Coronary Intervention**

**CABG：Coronary Artery Bypass Grafting**
